# Optimizing an existing prediction model for quality of life one‐year post‐intensive care unit: An exploratory analysis

**DOI:** 10.1111/aas.14138

**Published:** 2022-08-31

**Authors:** Manon de Jonge, Nina Wubben, Christiaan R. van Kaam, Tim Frenzel, Cornelia W. E. Hoedemaekers, Luca Ambrogioni, Johannes G. van der Hoeven, Mark van den Boogaard, Marieke Zegers

**Affiliations:** ^1^ Department Intensive Care Medicine Radboud University Medical Center, Radboud Institute for Health Sciences Nijmegen Netherlands; ^2^ Radboud University, Donders Institute for Brain, Cognition and Behaviour Nijmegen Netherlands

**Keywords:** critical care, machine learning, prediction modeling, quality of life, survivors

## Abstract

**Background:**

This study aimed to improve the PREPARE model, an existing linear regression prediction model for long‐term quality of life (QoL) of intensive care unit (ICU) survivors by incorporating additional ICU data from patients' electronic health record (EHR) and bedside monitors.

**Methods:**

The 1308 adult ICU patients, aged ≥16, admitted between July 2016 and January 2019 were included. Several regression‐based machine learning models were fitted on a combination of patient‐reported data and expert‐selected EHR variables and bedside monitor data to predict change in QoL 1 year after ICU admission. Predictive performance was compared to a five‐feature linear regression prediction model using only 24‐hour data (R^2^ = 0.54, mean square error (MSE) = 0.031, mean absolute error (MAE) = 0.128).

**Results:**

The 67.9% of the included ICU survivors was male and the median age was 65.0 [IQR: 57.0–71.0]. Median length of stay (LOS) was 1 day [IQR 1.0–2.0]. The incorporation of the additional data pertaining to the entire ICU stay did not improve the predictive performance of the original linear regression model. The best performing machine learning model used seven features (R^2^ = 0.52, MSE = 0.032, MAE = 0.125). Pre‐ICU QoL, the presence of a cerebro vascular accident (CVA) upon admission and the highest temperature measured during the ICU stay were the most important contributors to predictive performance. Pre‐ICU QoL's contribution to predictive performance far exceeded that of the other predictors.

**Conclusion:**

Pre‐ICU QoL was by far the most important predictor for change in QoL 1 year after ICU admission. The incorporation of the numerous additional features pertaining to the entire ICU stay did not improve predictive performance although the patients' LOS was relatively short.

**Editorial Comment**

In this sub‐study of the ongoing MONITOR‐IC study assessing long‐term outcomes after ICU survival, the authors assessed a subgroup from a single center. The findings were that the pre‐ICU QoL in this largely postsurgical cohort was by far the most important predictor for change in QoL 1 year after ICU admission.

## 1 INTRODUCTION

Annually, over 75,000 patients are admitted to Dutch intensive care units (ICUs). Due to advances in critical care medicine, more patients survive their critical illness.^1^ However, it is estimated that 25%–75% of ICU survivors experience long‐term physical, psychological, or cognitive complaints, as well as problems related to daily functioning.^2^, ^3^ These issues often negatively influence ICU survivors' quality of life (QoL) and financial and social situation. Consequently, ICU healthcare professionals' emphasis has shifted from not only mortality prevention but also to what surviving critical illness means for patients in the long term.

Currently, medical decisions about admission and treatment in the ICU are often based on the experience and intuition of ICU physicians to fill gaps in the knowledge of ICU physicians. Though this is undoubtedly important, ICU caregivers are not always able to reliably predict their patients' long‐term QoL^4^, ^5^, ^6^ and prognostic uncertainty may be a barrier for physicians for discussing long‐term ICU outcomes with patients and their family members.^7^ To better support ICU decision‐making and ameliorate the burden of prognostic uncertainty, the use of long‐term patient‐reported outcomes (PROMs) after ICU stay is of great relevance.^8^

Through the use of PROMs, prediction models that contain not only medical data but also patient‐reported data can be incorporated in ICU clinical decision making. There have only been two studies that investigate the prediction of long‐term QoL post‐ICU.^9^, ^10^ One of these is the PREPARE (PREdicting PAtients' long‐term outcome for REcovery) model^10^; a practically usable five‐variable prediction model for change in QoL 1 year after ICU admission. The PREPARE model has been developed by the authors of this study. Included predictors are pre‐ICU QoL and frailty status, sex, and ICU admission following a planned surgery and a cerebro‐vascular accident. Of these, pre‐ICU QoL was shown to be the most important contributor to the predictive performance of the PREPARE model.^10^ All of these predictors are measurable on the first day of ICU admission. The predictive performance of the model might be improved by the incorporation of additional patient data from the electronic health record (EHR) and bedside monitor data,^11^, ^12^ that pertains not only to the first day of ICU admission, but to the following days after admission as well. The further improvement may increase the validity and reliability of the model that could then be used to improve the quality of communication between physicians, patients and family members concerning long‐term ICU outcomes that takes place at the end of a patient's ICU stay.

Therefore, we aimed to improve the performance of the existing PREPARE prediction model^10^ for long‐term QoL by incorporating additional physiological, pathological, drug and treatment data from the EHR, including bedside monitor data, pertaining to the entire ICU stay. To facilitate the incorporation of the new data originating from several sources, machine learning methodology was used.

## 2 METHODS

### 2.1 Study design, setting, and population

This is a sub‐study of the MONITOR‐IC study, an ongoing multicenter prospective cohort study in which long‐term outcomes of ICU patients are measured up to 5 years after ICU admission (ClinicalTrials.gov: NCT03246334).^13^ The study protocol can be referred to for more detail.^13^ The MONITOR‐IC study has been approved by the research ethics committee of the Radboud University Medical Center (Radboudumc), CMO region Arnhem‐Nijmegen (number 2016–2724). Informed consent was obtained from all patients or their legal representative for the use of EHR data. All patient data were pseudo‐anonymized. This study uses single center data from a university hospital.

For this analysis, the follow‐up period was 1 year after ICU admission. Questionnaires were submitted at admission and 1 year after admission. Patients were excluded when they had been admitted to the ICU for less than 12 h, had a life expectancy of less than 48 h, or could not read or speak the Dutch language.

### 2.2 Data sources

The original prediction model (PREPARE model) was based on variables from the MONITOR‐IC database including patient‐reported data (e.g., QoL and frailty) and a limited number of variables from the EHR of the first 24‐hour of the ICU admission (e.g., admission type, diagnosis, severity of illness, and length of stay) (Additional File 1, Table S1). The PREPARE model was developed to predict change in QoL 1 year after ICU admission and is aimed at ICU‐survivors, to prepare them for life post their ICU admission. To build on the results of the first study, the same population was used in this study. During the development of the PREPARE model, multiple imputation was used to explore the consequences of independent variable missing data, but did not significantly change predictive performance and was subsequently not a part of the final methodology.^10^ As this study builds on the PREPARE model, multiple imputation was also not applied here.

In this study, the data used in the PREPARE model has been supplemented with additional EHR data (e.g., medication data, laboratory results and observations) and data from bedside monitors (e.g., blood pressure, body temperature) pertaining to the entire ICU stay. In order to do this, machine learning methodology was used.

Extracting the entire EHR database is expensive in terms of time and difficult regarding memory capacity. The EHR includes measurements that are likely of no influence to QoL and can be left out.^14^ Therefore, in order to narrow down the number of EHR variables, a series of expert meetings with four ICU physicians and one ICU nurse were organized. Within this process of expert‐based selection, variables that these clinicians know or suspect to have any influence on long‐term outcomes of QoL of ICU survivors were selected.^14^ This expert‐augmented machine learning approach narrows down the amount of training data, which reduces computational issues while keeping supposed influential factors in the data based on domain‐knowledge. In Additional File 1, Table S1, all selected variables are shown.

### 2.3 Primary outcome measure

The primary outcome measure is change in QoL: the index value on the EuroQol 5D (EQ‐5D‐5L) questionnaire 1 year after ICU admission minus the index value on the EQ‐5D‐5L questionnaire obtained at ICU admission pertaining to pre‐ICU health. The EQ‐5D‐5L is a standardized instrument for measuring QoL in medical care based on five questions. The Dutch EQ‐5D‐5L calibrated index values can range from −0.45 to 1, with higher index values indicating a better QoL.^15^

### 2.4 Preprocessing and feature selection of extended data

Preprocessing the data, selection of the most influential features and modeling the data were done in Python (version 3.7) using the scikit‐learn library.^16^ Outliers in the data were removed or corrected and missing values were replaced by group‐wide averages or zeros depending on the type of variable.^17^ The number of missing values per variable was never more than 5% of the total number of entries per variable. Group‐wide averages were used to replace missing values for bodily measurements and lab results. Missing values in medication data were replaced with zeros, as it could not be assumed that any medication was administered when no data was entered. Continuous physiological parameters (e.g., blood pressure) were preprocessed to represent the changes over time rather than the actual values.

To avoid overfitting and to increase the clarity and interpretability of the model, several features in the data were left out of the prediction model after discussions with medical professionals as well as artificial intelligence (AI) experts for reasons that included whether the medical professionals thought the variables of importance, data availability and available data quality. The selection process, as well as the variable derivatives chosen for each variable, can be viewed in Additional File 2, Table S2. Out of the remaining features, the most influential features were selected by recursively training on an ever‐shrinking set of features using Recursive Feature Elimination (RFE). This is a feature ranking method that recursively considers and assigns weights to smaller sets of features. The least important features were pruned. The feature set resulting in the highest adjusted R^2^ is used for modeling. RFE was not used in models that use regularization.

### 2.5 Prediction modeling

The PREPARE model was first replicated with machine learning techniques: the baseline model.^10^

Then, several machine learning techniques used for prediction were applied to the 24‐hour PREPARE data as well as to the data pertaining to the entire ICU stay in order to find the best performing prediction model. The 10 machine learning models that were compared are Ordinary Least Squares (OLS), Random Forest (RF) Regressor, Multilayer Perceptron (MLP) Regressor, Lasso(CV), Least Angle Regression (LARS), Elastic net regularization (ElasticNet), Huber Regressor, Ridge Regression, Automatic Relevance Determination (ARD) Regression, and Support Vector Regression (SVR) (Additional File 3, Table S3). Most of these models are linear models. The nonlinear models were used to explore whether they could fit the data more accurately than linear models.

The predictive performances of the different machine learning models using only 24‐hour PREPARE data were compared. Then, the best performing models were tweaked to perform optimally on the extended data set based on the entire stay.

### 2.6 Validation and evaluation

To be able to judge the machine learning models' performances and allow for direct comparison with the PREPARE model, the explained variance metric was used to evaluate the models after cross‐validation. Four‐fold cross validation was used for all models used in the current study. Another metric commonly used to evaluate regression models, the mean squared error (MSE), was also taken into consideration to provide a more complete overview of the results and to provide a comparison to the PREPARE model.^10^ The mean absolute error (MAE) was added as a metric in this study to give an indication of predictive performance. All these measures were also used to find the differences in performance between the models based on the data from the first 24 h of the ICU stay and the models based on data from the entire stay.

## 3 RESULTS

**FIGURE 1:**
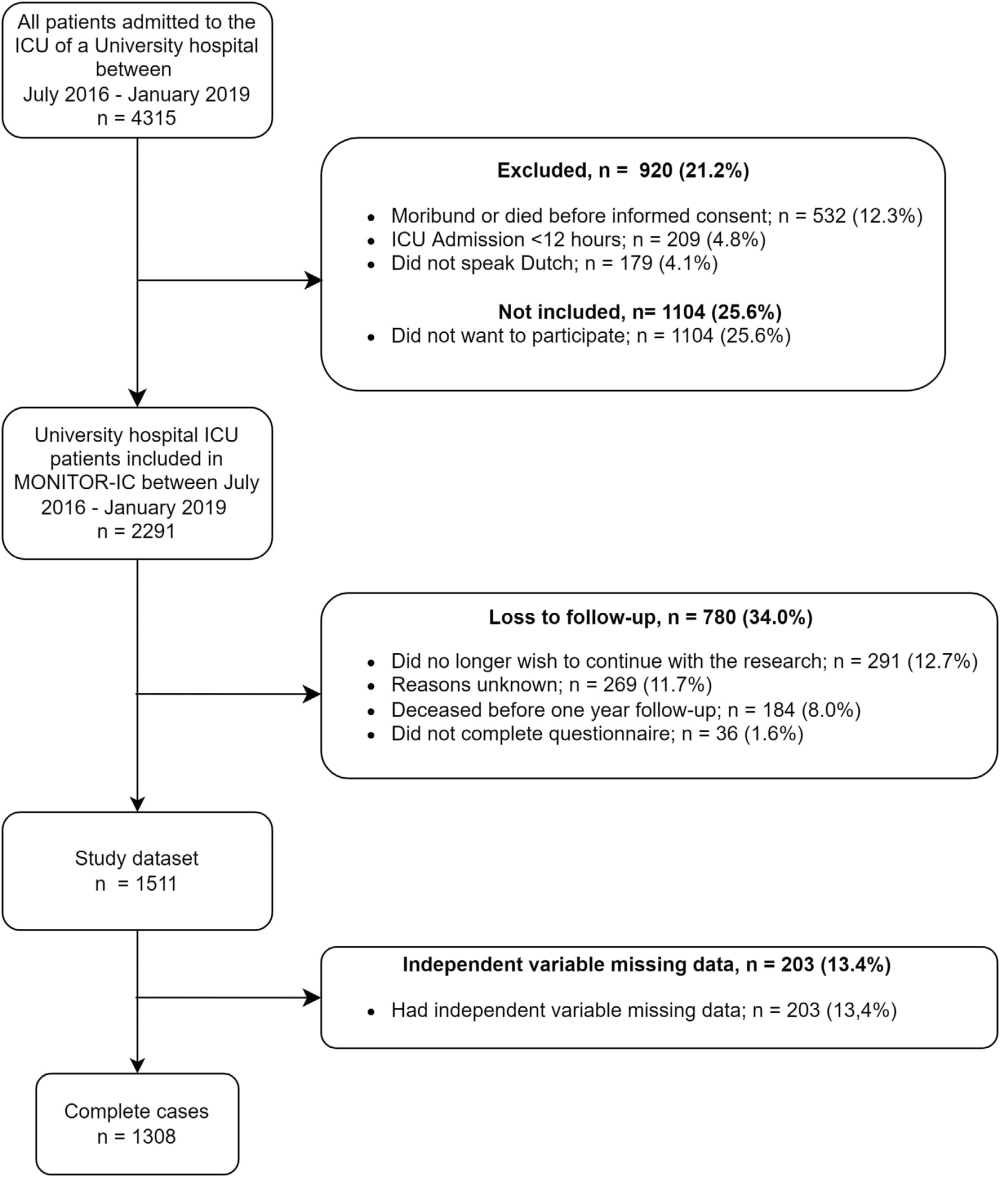
Study population flowchart

Between July 2016 and January 2019, a total of 4315 patients were admitted to the ICU, of whom 2291 patients were included in the MONITOR‐IC study. Of these, 780 (34.0%) were excluded because their one‐year outcome measurement was missing, either due to death (*n* = 184, 8.0%), a wish to discontinue study participation (*n* = 291, 12.7%), noncompletion of the questionnaire (*n* = 36, 1.6%) or other, unknown reasons (*n* = 269, 11.7%), resulting in a dataset of 1511 patients, of whom 1308 records had no other missing nonoutcome data (Figure 1). An overview of the baseline results for those who were lost to follow‐up due to nonsurvival or due to reasons other than nonsurvival (Additional File 4, Table S4) shows that those who were lost to follow‐up were more often female, had comparatively worse QoL index values at baseline and more chronic conditions.

### 3.1 Study population

The study group mainly consisted of male patients (67.9%). The median age of the patients was 65 years (first and third interquartile range (IQR) = 57–71). Most patients were admitted for planned surgery (72.7%). The median EQ‐5D‐5L index value before admission was 0.8 (IQR = 0.7–0.9). More characteristics of the study group are presented in Table 1.

**TABLE 1:** Patient demographics (*N* = 1308) at the time of ICU admission

Variable	Median [25–75%] or *N* (%)
Sex: male, *n* (%)	888 (67.9)
Age (years), median [IQR]	65.0 [57.0–71.0]
Frailty (CFS), median [IQR]	3 [2.0–3.0]
EQ‐5D‐5L index value, median [IQR]	0.8 [0.7–0.9]
Education level, *n* (%):	
High	376 (28.7)
Medium	574 (43.9)
Low	358 (27.4)
Comorbidity (chronic conditions), *n* (%):	
Immunological insufficiency	66 (5.0)
Malignant hematological disease	18 (1.4)
Metastasized neoplasm	58 (4.4)
Cirrhosis	0 (0)
Chronic cardiovascular insufficiency	37 (2.8)
Chronic respiratory insufficiency	16 (1.2)
Chronic renal insufficiency	21 (1.6)
Admission type, *n* (%):	
Planned surgery	951 (72.7)
Emergency surgery	140 (10.7)
Medical	217 (16.6)
APACHE IV score, median [IQR]	48.0 [38.0–60.0]
Mechanically ventilated within 24 h after admission, *n* (%)	1020 (78.0)
ICU length of stay (days)	1.0 [1.0–2.0]

Abbreviations: APACHE, acute physiology and chronic health evaluation; CFS, clinical frailty scale; EQ‐5D‐5L, EuroQoL‐5D‐5L.

### 3.2 Model performance based on 24‐hour data: Baseline model

To compare results with the PREPARE model, the methods used for validating PREPARE's five‐feature model were repeated. Adjusted R^2^, MSE and MAE were calculated over predictions from the entire data set. This resulted in an R^2^ of 0.54, an MSE of 0.031, and a MAE of 0.128. These scores differ very slightly from the PREPARE model's validation scores, which had an R^2^ of 0.55 and an MSE score of 0.030. These differences may be caused by the differences in algebra behind using Python versus R. The coefficients of the five predictors were similar in both models, with the QoL index value before ICU admission as the strongest predictor. Out of all predicted values, 48% differed less than 0.1 from their actual value and 82% differed less than 0.2.

### 3.3 Model performance based on 24‐hour data: Machine learning models

Next, the predictive performances of the different machine learning models were tested. The models were first developed using the 24‐hour data and consequently compared on their performance. Scores were calculated over predictions on unseen data. A more detailed view of the performances of the different machines learning models can be found in Additional File 3, Table S3. Based on MAE score, the best performing model for the 24‐hour data is the Huber Regressor. The model has an adjusted R^2^ of 0.52, an MSE of 0.032 and an MAE of 0.126.

### 3.4 Model performance based on entire ICU stay data: extended machine learning models

The variable selection process based on expert opinion can be viewed in Additional File 2, Table S2. EHR data are, in their original state, not suitable for modeling. After the selection process, the variables were therefore preprocessed and structured to fit the regression models.

The models were extended with 71 EHR data features (Additional File 5, Table S5) in addition to variables measured in the first 24‐hours of admission included in the PREPARE model. An overview of the features that remained after feature selection is presented in Table 2. Three features were selected in all models explored in this study: the baseline EQ‐5D‐5L index value, the presence/absence of a cerebrovascular accident (CVA), and the highest body temperature measured during a patient's stay. The first two are predictors from the PREPARE model, while the temperature measurement was an additional variable gathered from the EHR. Other features that are considered in most of the models are sex, frailty, low‐BMI scores, and delirium prevalence.

**TABLE 2:** The variables selected by RFE or a built‐in selection method based on feature importance

Variable	Selected by models^a^
EQ‐5D index value before admission	100%
Cerebro vascular accident (CVA)	100%
Highest temperature during ICU stay	100%
Sex	90%
Frailty before admission	80%
Low BMI	80%
Delirium prevalence	80%
Admission source	70%
Intracranial mass	70%
Respiratory insufficiency	70%
Low hemoglobin	60%
Malignant hematological disease	60%
Tracheostoma prevalence	40%
Nr. of registered mean inspiratory pressure (MIP) measurements	40%
Cardio pulmonary resuscitation (CPR)	40%
Dysrhythmia	40%
Low‐serum sodium	30%
Mechanically ventilated in the first 24 h	10%
Length of stay (LOS)	10%

^a^ Selected by RFE or built‐in regularization, models that have no built‐in feature importance metric were tested by hand. Inclusion in all of the 10 compared models equals 100%.

The number of features selected per model after the addition of more EHR data ranges from 7 to 15. In every model, the addition led to slightly improved adjusted R^2^, MSE and/or MAE scores. Improved adjusted R^2^ scores differed in a range of 0%–5% from the five‐feature baseline model. MSE improvements reached a maximum difference of 0.003. Every model had a lower MAE using the additional EHR features when compared to the classic model, with the lowest MAE being 0.125 in the Huber Regressor and SVR models. The Huber Regressor model was (among the) best performing model(s) when using both 24‐hour and extended data.

A direct comparison of model predictive performance after the addition of more EHR and bedside monitor data should only be made for models that use the same methodology (Table 3). Therefore, comparatively, the Huber Regressor model using extended data, which had an R^2^ of 0.52, an MSE of 0.032, and a MAE of 0.125, delivered an equal R^2^ and MSE and a very slightly improved MAE when compared to the Huber Regressor model that used only 24‐hour data. Though a direct comparison with the performance of the baseline model, the classical prediction model replicating using machine learning techniques, cannot be made, its performance overall appears similar as well. Of the three perpetually selected features, baseline (pre‐ICU) EQ‐5D‐5L QoL index value was the most important, as it contributed most to a reduction in the MAE (results not shown).

**TABLE 3:** Features included and predictive performance of the Huber Regressor model using 24‐hour data compared to the Huber Regressor model using data pertaining to the whole ICU stay

Variables selected	By 24‐hour model	By extended data model
	EQ5D index value before admission	EQ5D index value before admission
	Cerebro vascular accident (CVA)	Cerebro vascular accident (CVA)
	ICU admission followed planned surgery	Highest temperature during ICU stay*
	Sex	Sex
	Frailty score before admission	Frailty score before admission
		Low BMI^a^
		Delirium prevalence^a^
Predictive performance		
R^2^	0.54	0.52
MSE	0.031	0.032
MAE	0.128	0.125

Abbreviations: MSE, mean squared error; MAE, mean absolute error; R^2^, explained variance.

^a^ Features introduced in the extended dataset using EHR and bedside monitoring data.

The changes in QoL index values can theoretically range from −1.45 to 1.45 due to the way in which the Dutch EQ‐5D‐5L scoring method is defined.^15^ In this study, the minimum change in QoL was −1.2 and the maximum change was 1.3. The IQR of the change in QoL was −0.07–0.15. For the Huber Regressor, 53% of the predictions differ less than 0.1 from their actual value and 84% differ less than 0.2. This means that a shift from 5 to 7 features leads to increases of 5% and 2% in predictive performance respectively in comparison to the classic linear regression model.

**FIGURE 2:**
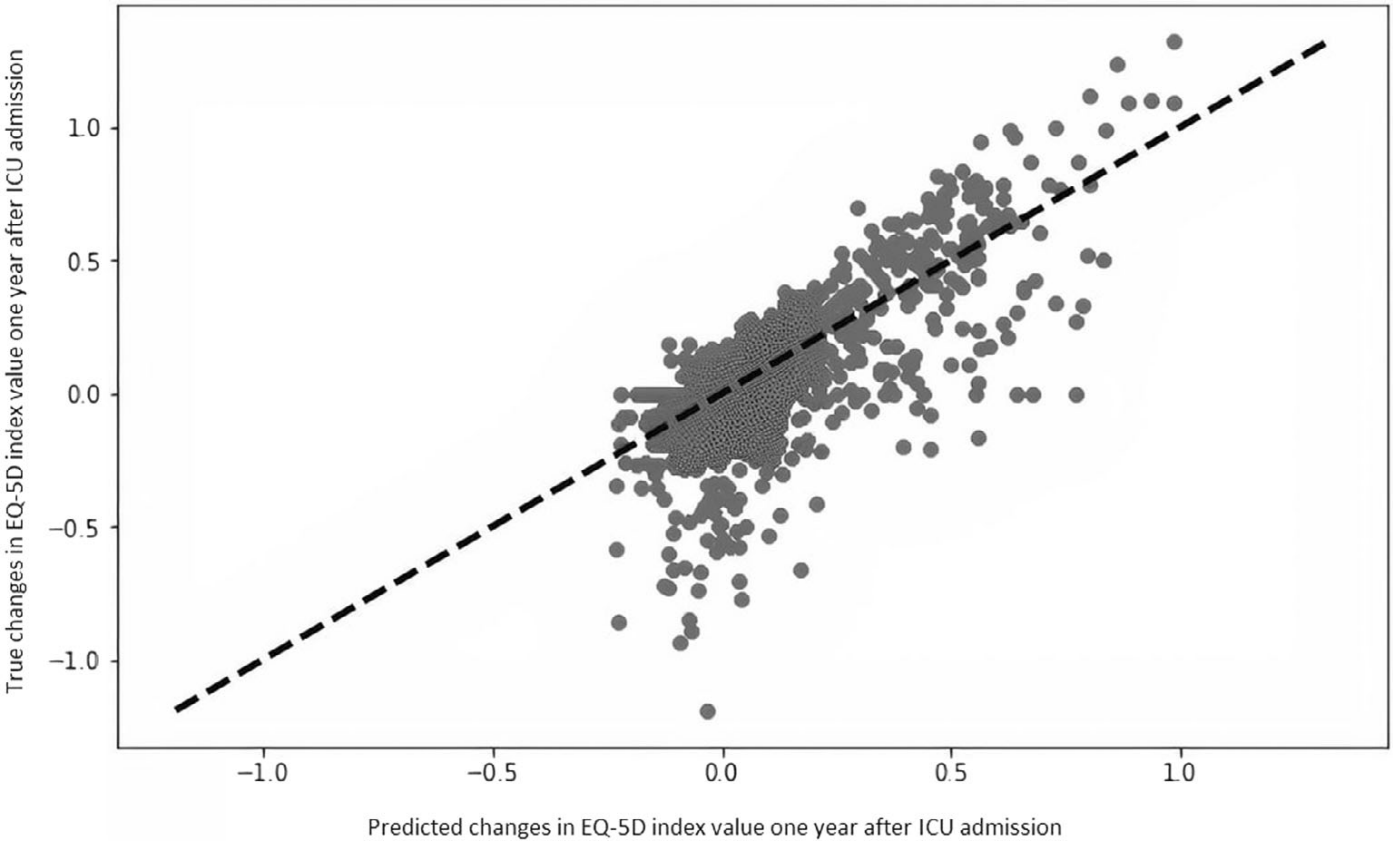
Calibration plot for the predictions of the Huber Regressor model with seven features

In Figure 2, the predictions of the Huber Regressor with seven features are plotted against the true values for change in QoL. The plot shows that the model does not accurately predict the lower negative and the highest positive QoL changes, while it performs passably for the other values. This pattern can be seen for all the models compared in this study.

## 4 DISCUSSION

This study showed that overall, pre‐ICU QoL was by far the most important predictor for change in QoL 1 year after ICU‐admission. This result was also seen during the development of the existing PREPARE model that used data of the first 24 h of admission.^10^ The incorporation of the additional expert‐selected EHR and bedside monitoring data pertaining to the entire ICU‐stay did not change this, nor did it improve model performance. Like the original PREPARE prediction model and previous literature,^10^, ^18^, ^19^, ^20^, ^21^ this study showed that the most important predictor for change in long‐term QoL was the QoL before admission. For ICU survivors, QoL 1 year after ICU admission is largely determined by pre‐ICU QoL.^10^ Even though the aim of this study, like the previous study about the development of the PREPARE model,^10^ was not to identify and explain the associations found, our finding that pre‐ICU QoL is strongly associated with long‐term QoL and other long‐term health outcomes is reflected in other literature.^9^, ^18^, ^19^, ^22^ A patient's pre‐ICU health status appears to be an important indicator for their post‐ICU outcomes and should play a part in ICU decision‐making and communication with patients and family members. Its importance as a predictor was such that the straightforward addition of available EHR and bedside monitoring measurements made during ICU stay do not meaningfully improve the predictive performance for a prediction model for change in QoL 1 year after ICU admission. Aside from pre‐ICU QoL, high temperatures, low BMI, and delirium prevalence were some of the variables rated as important by the machine‐learning models in the present study. Associations between QoL and these features have been found in previous studies.^23^, ^24^, ^25^ More research is needed to evaluate the effects of these and other influential EHR variables on patients' QoL changes after ICU admission.

As prognostic uncertainty fosters inaccurate expectations for future recovery and may decrease the quality of ICU communication, optimizing the use of long‐term outcome data in ICU practice is of the utmost importance,^26^, ^27^, ^28^ especially since many former ICU patients still experience health problems a year after their admission.^3^ This is the first study in which data pertaining to the entirety of an ICU patient's admission were used to predict changes in long‐term QoL. As mentioned, the methods used to incorporate the additional data come from the field of machine learning. Previous studies that have employed such methods for both short‐ and long‐term prediction in medicine^29^, ^30^, ^31^ showed an increase in predictive performance after the addition of EHR data. Others^32^, ^33^, ^34^, ^35^ have shown that machine learning is capable of finding associations between patient characteristics (e.g., substance abuse and/or socioeconomic status) and certain medical conditions also related to QoL. The importance of pre‐ICU QoL as a predictor, compared to the other predictors used in this study, might explain our finding of no improvement of predictive performance.^10^, ^18^

This study has some limitations. First, over two thirds of the study population were included after elective surgery. As these patients generally have better outcomes after their ICU‐admission and are admitted for a shorter time, this sample may therefore reflect a slightly healthier cohort than the general ICU survivor population, that may have lower odds of ICU events that might have an influence on QoL 1 year after ICU admission. Also, the relatively short length of stay in our population will likely have decreased the time in which events chronicled by EHR or bedside monitor data could have an impact on long‐term QoL. Second, within our methodology, compared to datasets generally used in machine learning, our population size was limited. Machine learning models generally need large amounts of training data to reach a reliable prediction result.^33^, ^35^ Also, approximately a third of the patients were lost to follow‐up. An overview of the baseline results for the group included the analyses compared to those who were lost to follow‐up due to nonsurvival or reasons other than nonsurvival (Additional File 4, Table S4) shows that those that were lost to follow‐up had comparatively worse QoL index values at baseline and more chronic conditions than the group included in the analyses. This nonresponse bias again may have caused our sample to reflect a slightly healthier cohort. However, it should be noted that the same approach with regards to missing values was taken during the development of the PREPARE model. This loss‐to‐follow‐up has therefore not influenced the comparative performances of the PREPARE prediction model and the prediction models developed in this study.

In future research, the analysis could be replicated in a larger patient group, with a higher proportion of ICU patients with a medical admission and with a larger variety in length of stay, to verify the results. Furthermore, several expert‐selected variables were not included in this study as the data for these variables could not be extracted from the EHR database.^30^ Also, the derived features from the extracted variables might not properly represent the consequences for the changes in QoL.^36^ However, as pre‐ICU QoL topped all other bedside monitor and EHR variables in terms of predictive importance, the influence of these omissions is not estimated to be large.

All in all, pre‐ICU QoL has high‐predictive value in predicting long‐term QoL.^10^, ^18^ Our findings here suggest that the incorporation of pre‐ICU QoL information in prediction models for long‐term QoL renders the inclusion of more comprehensive physiological data unnecessary. Therefore, future implementation of a prediction model for long‐term QoL in critical care practice to improve the quality of communication between physicians, patients and family members about long‐term ICU outcomes might be able to be achieved using the comparatively less complicated 5‐feature linear regression PREPARE model.

## 5 CONCLUSION

This study showed that pre‐ICU QoL was by far the most important predictor for change in QoL 1 year after ICU‐admission in a prediction model that used data pertaining to the entire ICU stay. The incorporation of readily available additional straightforward expert‐selected EHR and bedside monitor measurements pertaining to the entire ICU stay did not improve the predictive performance of an existing prediction model using only data from the first 24 h of admission, although it is important to note that the length of stay of this population was relatively short. Future research might improve the predictive performance by employing a larger cohort and more expert‐selected predictors. Until then, the implementation of QoL outcomes into critical care practice might be able to be achieved using a comparatively less complicated model that is understandable for clinicians.

## FUNDING INFORMATION

This work was supported by the National Health Care Institute (2018026879). The National Health Care Institute was not involved in the design of the study, nor with the data collection, analysis, interpretation or writing of the manuscript.

## CONFLICT OF INTEREST

The author declares there is no potential conflicts of interest.

## Supporting information


**Table S1** Variable preselection for PREPARE from the first 24 h of admission based on expert opinion [10]

**Table S2** Variable selection process

**Table S3** Prediction scores per model after cross‐validation

**Table S4** Characteristics of complete cases, patient who were lost to follow up due to nonsurvival and those lost to follow up due to other reasons

**Table S5** An overview of the expert‐selected variables for QoL prediction

## Data Availability

The datasets used and analyzed in this study are available from the corresponding author on reasonable request.

## References

[1] Makic MB . Recovery after ICU discharge: post‐intensive care syndrome. J Perianesth Nurs. 2016;31(2):172‐174. 27037171 10.1016/j.jopan.2015.12.006

[2] Svenningsen H , Egerod I , Christensen D , Tønnesen EK , Frydenberg M , Videbech P . Symptoms of posttraumatic stress after intensive care delirium. Biomed Res Int. 2015;2015:876947. 26557708 10.1155/2015/876947 PMC4628708

[3] Geense WW , Zegers M , Peters MAA , et al. New physical, mental, and cognitive problems 1‐year post‐ICU: a prospective multicenter study. Am J Respir Crit Care Med. 2021;203:1512‐1521. 33526001 10.1164/rccm.202009-3381OC

[4] Frick S , Uehlinger DE , Zuercher Zenklusen RM . Medical futility: predicting outcome of intensive care unit patients by nurses and doctors–a prospective comparative study. Crit Care Med. 2003;31(2):456‐461. 12576951 10.1097/01.CCM.0000049945.69373.7C

[5] Kerckhoffs MC , Kosasi FFL , Soliman IW , et al. Determinants of self‐reported unacceptable outcome of intensive care treatment 1 year after discharge. Intensive Care Med. 2019;45(6):806‐814. 30840124 10.1007/s00134-019-05583-4 PMC6534510

[6] Soliman IW , Cremer OL , de Lange DW , et al. The ability of intensive care unit physicians to estimate long‐term prognosis in survivors of critical illness. J Crit Care. 2018;43:148‐155. 28898744 10.1016/j.jcrc.2017.09.007

[7] Turnbull AE , Davis WE , Needham DM , White DB , Eakin MN . Intensivist‐reported facilitators and barriers to discussing post‐discharge outcomes with intensive care unit surrogates. A qualitative study. Ann Am Thorac Soc. 2016;13(9):1546‐1552. 27294981 10.1513/AnnalsATS.201603-212OC PMC5059504

[8] Needham DM , Davidson J , Cohen H , et al. Improving long‐term outcomes after discharge from intensive care unit: report from a stakeholders' conference. Crit Care Med. 2012;40(2):502‐509. 21946660 10.1097/CCM.0b013e318232da75

[9] Oeyen S , Vermeulen K , Benoit D , Annemans L , Decruyenaere J . Development of a prediction model for long‐term quality of life in critically ill patients. J Crit Care. 2018;43:133‐138. 28892669 10.1016/j.jcrc.2017.09.006

[10] Wubben N , van den Boogaard M , Ramjith J , et al. Development of a practically usable prediction model for quality of life of ICU survivors: a sub‐analysis of the MONITOR‐IC prospective cohort study. J Crit Care. 2021;65:76‐83. 34111683 10.1016/j.jcrc.2021.04.019

[11] Shukla SN , Marlin BM . Integrating physiological time series and clinical notes with deep learning for improved ICU mortality prediction. ACM Conf Health Inference Learn. 2020.

[12] Tonekaboni S , Mazwi M , Laussen P , et al. Prediction of cardiac arrest from physiological signals in the pediatric ICU. Proc 3rd Mach Learn Healthcare Conf. 2018;85:534‐550.

[13] Geense W , Zegers M , Vermeulen H , van den Boogaard M , van der Hoeven J . MONITOR‐IC study, a mixed methods prospective multicentre controlled cohort study assessing 5‐year outcomes of ICU survivors and related healthcare costs: a study protocol. BMJ Open. 2017;7(11):e018006. 10.1136/bmjopen-2017-018006 PMC5695418 29138206

[14] Gennatas ED , Friedman JH , Ungar LH , et al. Expert‐augmented machine learning. Proc Natl Acad Sci U S A. 2020;117(9):4571‐4577. 32071251 10.1073/pnas.1906831117 PMC7060733

[15] Versteegh M , Vermeulen K , Evers S , de Wit GA , Prenger RES . Dutch tariff for the five‐level version of EQ‐5D. Value Health. 2016;19(4):343‐352. 27325326 10.1016/j.jval.2016.01.003

[16] Pedregosa FVG , Gramfort A , Michel V , Thirion B , Grisel O . Scikit‐learn: machine learning in python. J Mach Learn Res. 2011;12(85):2825‐2830.

[17] Eekhout I , de Boer RM , Twisk JW , de Vet HC , Heymans MW . Missing data: a systematic review of how they are reported and handled. Epidemiology. 2012;23(5):729‐732. 22584299 10.1097/EDE.0b013e3182576cdb

[18] Cuthbertson BH , Wunsch H . Long‐term outcomes after critical illness. The best predictor of the future is the past. Am J Respir Crit Care Med. 2016;194(2):132‐134. 26953728 10.1164/rccm.201602-0257ED

[19] Hofhuis JG , Spronk PE , van Stel HF , Schrijvers AJ , Bakker J . Quality of life before intensive care unit admission is a predictor of survival. Crit Care. 2007;11(4):R78. 17629906 10.1186/cc5970 PMC2206516

[20] Pietiläinen L , Hästbacka J , Bäcklund M , Parviainen I , Pettilä V , Reinikainen M . Premorbid functional status as a predictor of 1‐year mortality and functional status in intensive care patients aged 80 years or older. Intensive Care Med. 2018;44(8):1221‐1229. 29968013 10.1007/s00134-018-5273-y

[21] Milton A , Schandl A , Soliman I , et al. ICU discharge screening for prediction of new‐onset physical disability‐a multinational cohort study. Acta Anaesthesiol Scand. 2020;64(6):789‐797. 32083323 10.1111/aas.13563

[22] Orwelius L , Nordlund A , Nordlund P , et al. Pre‐existing disease: the most important factor for health related quality of life long‐term after critical illness: a prospective, longitudinal, multicentre trial. Crit Care. 2010;14(2):R67. 20398310 10.1186/cc8967 PMC2887189

[23] Fontaine KR , Barofsky I . Obesity and health‐related quality of life. Obes Rev. 2001;2(3):173‐182. 12120102 10.1046/j.1467-789x.2001.00032.x

[24] Matterne U , Schmitt J , Diepgen TL , Apfelbacher C . Children and adolescents' health‐related quality of life in relation to eczema, asthma and hay fever: results from a population‐based cross‐sectional study. Qual Life Res. 2011;20(8):1295‐1305. 21347571 10.1007/s11136-011-9868-9

[25] van den Boogaard M , Schoonhoven L , Evers AW , van der Hoeven JG , van Achterberg T , Pickkers P . Delirium in critically ill patients: impact on long‐term health‐related quality of life and cognitive functioning. Crit Care Med. 2012;40(1):112‐118. 21926597 10.1097/CCM.0b013e31822e9fc9

[26] Daly BJ , Douglas SL , Lipson AR . Family and nurse prognostication in chronic critical illness. Int J Nurs Res. 2018;4(4):281‐287. 31098418 PMC6516068

[27] Douglas SL , Daly BJ , Lipson AR . Differences in predictions for survival and expectations for goals of care between physicians and family surrogate decision makers of chronically critically ill adults. Res Rev J Nurs Health Sci. 2017;3(3):74‐84. 29911208 PMC6003707

[28] Cox CE , Martinu T , Sathy SJ , et al. Expectations and outcomes of prolonged mechanical ventilation. Crit Care Med. 2009;37(11):2888‐2894. quiz 904. 19770733 10.1097/CCM.0b013e3181ab86ed PMC2766420

[29] Chiew CJ , Liu N , Tagami T , Wong TH , Koh ZX , Ong MEH . Heart rate variability based machine learning models for risk prediction of suspected sepsis patients in the emergency department. Medicine (Baltimore). 2019;98(6):e14197. 30732136 10.1097/MD.0000000000014197 PMC6380871

[30] Roimi M , Neuberger A , Shrot A , Paul M , Geffen Y , Bar‐Lavie Y . Early diagnosis of bloodstream infections in the intensive care unit using machine‐learning algorithms. Intensive Care Med. 2020;46(3):454‐462. 31912208 10.1007/s00134-019-05876-8

[31] Wijnberge M , Geerts BF , Hol L , et al. Effect of a machine learning‐derived early warning system for intraoperative hypotension vs standard care on depth and duration of intraoperative hypotension during elective noncardiac surgery: the HYPE randomized clinical trial. Jama. 2020;323(11):1052‐1060. 32065827 10.1001/jama.2020.0592 PMC7078808

[32] Davoudi A , Ebadi A , Rashidi P , Ozrazgat‐Baslanti T , Bihorac A , Bursian AC . Delirium prediction using machine learning models on preoperative electronic health records data. Proc IEEE Int Symp Bioinfo Bioeng. 2017;2017:568‐573. 10.1109/BIBE.2017.00014 PMC6211171 30393788

[33] Khan O , Badhiwala JH , Witiw CD , Wilson JR , Fehlings MG . Machine learning algorithms for prediction of health‐related quality‐of‐life after surgery for mild degenerative cervical myelopathy. Spine J. 2020;21:1659‐1669. 32045708 10.1016/j.spinee.2020.02.003

[34] Miotto R , Li L , Kidd BA , Dudley JT . Deep patient: an unsupervised representation to predict the future of patients from the electronic health records. Sci Rep. 2016;6:26094. 27185194 10.1038/srep26094 PMC4869115

[35] Sim JA , Kim YA , Kim JH , et al. The major effects of health‐related quality of life on 5‐year survival prediction among lung cancer survivors: applications of machine learning. Sci Rep. 2020;10(1):10693. 32612283 10.1038/s41598-020-67604-3 PMC7329866

[36] Wu J , Roy J , Stewart WF . Prediction modeling using EHR data: challenges, strategies, and a comparison of machine learning approaches. Med Care. 2010;48(6 Suppl):S106‐S113. 20473190 10.1097/MLR.0b013e3181de9e17

