# Supplementary material for: Optimizing an existing prediction model for quality of life one‐year post‐intensive care unit: An exploratory analysis
Source: Acta Anaesthesiol Scand. 2022 Aug 31;66(10):1228–36. doi: 10.1111/aas.14138 (PMC9804831; doi:10.1111/aas.14138)
Supplement: Supplementary file 1 — Table S1 Variable preselection for PREPARE from the first 24 h of admission based on expert opinion [10] [file AAS-66-1228-s005.docx]

*Additional File 1:* Overview of the variables available and/or selected for analysis (Table 1).

**Table S1.** Variable preselection for PREPARE from the first 24 hours of admission based on expert opinion [10]

| **Available variable** | **Selected for analysis** |
| --- | --- |
| *PROMs describing health situation before admission unless specified otherwise* | |
| Age |  |
| Sex |  |
| Height |  |
| Weight |  |
| Nationality |  |
| Marital status |  |
| Education |  |
| *PROMs describing health situation and other variables before admission unless specified otherwise* | |
| Clinical Frailty Score (CFS) |  |
| SF-36 PCS score |  |
| SF-36 MCS score |  |
| Checklist Individual Strength (CIS) |  |
| Baseline EQ-5D-5L score |  |
| EQ-5D Visual Analogue Scale (VAS) |  |
| Hospital Anxiety and Depression Scale (HADS) |  |
| Source of income |  |
| Previous admission(s) to the ICU |  |
| Cognitive failure questionnaire (CFQ) |  |
| *Electronic health record data, measured at admission or within the first 24 hours of admission* | |
| BMI |  |
| Referring specialism |  |
| Admission source |  |
| Admission type |  |
| Cardio Pulmonary Resuscitation (CPR) |  |
| Acute renal failure (originating on ICU) |  |
| Dysrhythmia |  |
| Cerebrovascular accident (CVA) (present at admission or within the first hour of admission) |  |
| Intracranial mass (present at admission or within the first hour of admission) |  |
| Chronic renal insufficiency |  |
| Chronic dialysis |  |
| Metastasized neoplasm (previously established) |  |
| AIDS |  |
| Malignant hematological disease (previously established) |  |
| Cirrhosis |  |
| Chronic cardiovascular insufficiency |  |
| Chronic respiratory insufficiency |  |
| Immunological insufficiency (previously established) |  |
| Glasgow Coma Score (GCS) at admission |  |
| GCS after first 24 hours of admission |  |
| Minimum GCS in first 24 hours of admission |  |
| APACHE II admission diagnosis category |  |
| Heartrate at admission |  |
| Minimum heartrate in first 24 hours |  |
| Maximum heartrate in first 24 hours |  |
| Minimum respiratory rate in first 24 hours |  |
| Maximum respiratory rate in first 24 hours |  |
| Systolic blood pressure at IC admission |  |
| Minimum systolic blood pressure in first 24 hours |  |
| Maximum systolic blood pressure in first 24 hours |  |
| Minimum mean blood pressure in first 24 hours |  |
| Maximum mean blood pressure in first 24 hours |  |
| Minimum body temperature in first 24 hours |  |
| Maximum body temperature in first 24 hours |  |
| PTT |  |
| INR |  |
| Urine output in first 24 hours |  |
| Vaso-active medication |  |
| PaCO_2_ |  |
| FiO_2_ |  |
| PaO_2_ |  |
| Minimum pH |  |
| Minimum white blood cell count |  |
| Maximum white blood cell count |  |
| Minimum creatinine level in first 24 hours |  |
| Maximum creatinine level in first 24 hours |  |
| Minimum potassium level in first 24 hours |  |
| Maximum potassium level in first 24 hours |  |
| Minimum sodium level in first 24 hours |  |
| Maximum sodium level in first 24 hours |  |
| Minimum bicarbonate level in first 24 hours |  |
| Maximum bicarbonate level in first 24 hours |  |
| Serum urea |  |
| Maximum bilirubin level in first 24 hours |  |
| Minimum hematocrit level in first 24 hours |  |
| Maximum hematocrit level in first 24 hours |  |
| Minimum hemoglobin level in first 24 hours |  |
| Maximum hemoglobin level in first 24 hours |  |
| Minimum albumin level in first 24 hours |  |
| Maximum albumin level in first 24 hours |  |
| Minimum thrombocyte level in first 24 hours |  |
| Minimum glucose level in first 24 hours |  |
| Maximum glucose level in first 24 hours |  |
| Confirmed infection |  |
| Mechanically ventilated at admission |  |
| Mechanically ventilated within 24 hours of admission |  |
| Mortality Prediction Model (MPM) at admission |  |
| MPM after 24 hours |  |
| Simplified acute physiology score (SAPS) score in first 24 hours |  |
| SAPS probability score in first 24 hours |  |
| Acute physiology and chronic health evaluation (APACHE) score |  |
| APACHE probability score in first 24 hours |  |
| APACHE IV score in first 24 hours |  |
| APACHE IV probability score in first 24 hours |  |
| Burns |  |
| Sepsis |  |
| Thrombolytic therapy after acute myocardial infarction |  |
| Internal Mammary Arterial graft |  |
| COPD |  |
| Diabetes |  |
| Myocardial infarction before ICU admission |  |
| Critical illness polyneuropathy |  |
| *Electronic health record data, measured during entirety of admission* | |
| Decubitus |  |
| Digestive tract bleeding |  |
| Cardiac arrest (minimally 4 hours after admission) |  |
| CVA during ICU admission |  |
| Trachea tube present |  |
| Maximum SOFA score |  |
