# Supplementary material for: Optimizing an existing prediction model for quality of life one‐year post‐intensive care unit: An exploratory analysis
Source: Acta Anaesthesiol Scand. 2022 Aug 31;66(10):1228–36. doi: 10.1111/aas.14138 (PMC9804831; doi:10.1111/aas.14138)
Supplement: Supplementary file 2 — Table S2 Variable selection process [file AAS-66-1228-s001.docx]

*Additional File 2:* Variable selection process

**Table S2.** Variable selection process

| Variable category and name* | | EHR data extraction | In final selection | Variable derivatives |
| --- | --- | --- | --- | --- |
| Lab | Hemoglobin | Blood gas, Point-of-care testing (POCT) | Yes (blood gas) | Minimum, times values were below 3, 4, 5 and 6 mmol/L, std dev |
|  | Glucose | Blood gas, liquor, urine, POCT | Yes (blood gas) | Minimum, maximum, values below 4 mmol/L, values above 8 mmol/L, std dev |
|  | pH | Blood gas, urine, POCT | No |  |
|  | Sodium | Blood gas, urine, POCT | Yes (blood gas) | Minimum, maximum, values below 130 mEq/L, std dev |
|  | Creatinine | Urine | No |  |
|  | Lactate | Blood gas | Yes | Maximum, values above 2.1 mmol/L, std dev |
| Medication | Dobutamine | IV drip (several doses) | No |  |
|  | Midalozam | IV drip (several doses), bolus, nose spray, tablet | Yes (IV drip, bolus tablet) | Cumulative (relative to LOS)**, nr. days medicated |
|  | Milrinon | IV drip (several doses) | No |  |
|  | Noradrenaline | IV drip (several doses), ampoule | Yes | Cumulative (relative to LOS)**, nr. days medicated |
|  | Propofol | IV drip (10 mg/ml), injection | Yes | Cumulative (relative to LOS)**, nr. days medicated |
|  | Rocoronium | IV drip (several doses), injection | No |  |
|  | Vasopressine | IV drip (1 unit/ml in NaCl) | No |  |
| Measurements | Temperature | °C, measurement location | Yes (°C) | Minimum, maximum, values below 35.5°C , values above 38.3 °C, std dev |
|  | Fraction of inspired oxygen (FiO2) | Oxygen percentage | Yes | Minimum, maximum, std dev, average per day*** |
|  | Intracranial pressure (ICP) | mmHg | Yes | Boolean (measured/not measured) |
|  | Extra corporeal life support (ECLS) | L/min | No |  |
|  | Positive end-expiratory pressure (PEEP) | Pressure (cm/H20) | Yes | Minimum, maximum, std dev, average per day*** |
|  | Glasgow coma scale (GCS) | Eye opening, motor response, verbal response | No |  |
| Raw (monitor data) | Heart rate | Beats per minute | Yes | Minimum, maximum, std dev, average per day*** |
|  | Blood pressure | Systolic, diastolic, mean | Yes (mean) | Minimum, maximum, std dev, average per day*** |
|  | Respiratory rate | Respirations per minute | No |  |
| Other | Tracheostoma | Tracheacanule prevalence | Yes | Boolean (measured/not measured), nr. of times |
|  | Renal replacement therapy (RRT) | RRT prevalence | Yes | Boolean (measured/not measured), nr. of times |
|  | Fluid balance | Day balance | No |  |
|  | Delirium | Subtype, prevalence, comatose days | Yes (prevalence, comatose days) | Boolean (measured/not measured), nr. of times |
|  | Length of stay (LOS) | Admission date and time, discharge date and time | Yes | Nr. of hours |
|  | Timing of admission | Admission date and time | Yes | Boolean (within/outside office hours) |

* Several expert-selected variables are not included in this overview as the data for these variables could not be extracted directly from the EHR database. These variables include: ventilator-associated pneumonia (VAP), catheter-related infection (CRI), bacteraemia, percutane drainage abcess, tractus digestivus bleeding, new ICU bleeding, ICU acquired weakness, decompressive craniectomy, andcardiac arrest. As experts have suggested the use of these variables, they could be included in future research to test their effect on QoL prediction.
** The cumulative dose divided by ICU LOS.
*** Derivatives used are minimum value, maximum value, and standard deviation across the entire ICU stay as well as the average, (calculated by first calculating the average per day, and then by taking these measurements and calculating the average for the entire stay.

Abbreviations: POCT = Point Of Care Testing; LOS = Length of Stay; FiO_2_ = Fraction of inspired oxygen; ICP = Intracranial Pressure; ECLS = Extra Corporeal Life Support; PEEP = Positive End-Expiratory Pressure; GCS = Glasgow Coma Scale; RRT = Renal Replacement Therapy
