# Supplementary material for: Optimizing an existing prediction model for quality of life one‐year post‐intensive care unit: An exploratory analysis
Source: Acta Anaesthesiol Scand. 2022 Aug 31;66(10):1228–36. doi: 10.1111/aas.14138 (PMC9804831; doi:10.1111/aas.14138)
Supplement: Supplementary file 3 — Table S3 Prediction scores per model after cross‐validation [file AAS-66-1228-s004.docx]

*Additional File 3.* Prediction scores per model after cross-validation

Table S3. Prediction scores per model after cross-validation

|  | *Five-feature 24-hour data* | | | *Extended data, consisting of baseline, additional EHR data and bedside data* | | | |
| --- | --- | --- | --- | --- | --- | --- | --- |
| Model | adj. R^2^ | MSE | MAE | Nr. of features | adj.R^2^ | MSE | MAE |
| OLS | 0.52 | 0.032 | 0.129 | 9 | 0.54 | 0.031 | 0.127 |
| RF Regressor | 0.51 | 0.033 | 0.130 | 7 | 0.51 | 0.032 | 0.129 |
| MLP Regressor | 0.46 | 0.035 | 0.136 | 11 | 0.51 | 0.032 | 0.127 |
| LassoCV | 0.52 | 0.032 | 0.129 | 8 | 0.53 | 0.031 | 0.128 |
| LARS | 0.52 | 0.032 | 0.129 | 13 | 0.54 | 0.030 | 0.127 |
| ElasticNet | 0.52 | 0.032 | 0.129 | 13 | 0.54 | 0.030 | 0.127 |
| Huber Regressor | 0.52 | 0.032 | 0.126 | 7 | 0.52 | 0.032 | 0.125 |
| Ridge Regression | 0.52 | 0.032 | 0.129 | 13 | 0.54 | 0.030 | 0.127 |
| ARD Regression | 0.52 | 0.032 | 0.129 | 15 | 0.53 | 0.031 | 0.127 |
| SVR | 0.52 | 0.032 | 0.127 | 13 | 0.53 | 0.031 | 0.125 |

Abbreviations: adj. R^2^ = adjusted R^2^; MSE = Mean Square Error; MAE = Mean Absolute Error
