# Supplementary material for: Optimizing an existing prediction model for quality of life one‐year post‐intensive care unit: An exploratory analysis
Source: Acta Anaesthesiol Scand. 2022 Aug 31;66(10):1228–36. doi: 10.1111/aas.14138 (PMC9804831; doi:10.1111/aas.14138)
Supplement: Supplementary file 4 — Table S4 Characteristics of complete cases, patient who were lost to follow up due to nonsurvival and those lost to follow up due to other reasons [file AAS-66-1228-s003.docx]

*Additional File 4*. Characteristics of complete cases, patient who were lost to follow up due to non-survival and those lost to follow up due to other reasons

**Table S4.** Characteristics of complete cases, patient who were lost to follow up due to non-survival and those lost to follow up due to other reasons

| Variable | Complete cases  (n=1308) | Lost to follow up due to non-survival (n=184) | P-value | Lost to follow up due to other reason (n=596) | P-value |
| --- | --- | --- | --- | --- | --- |
| Sex: male, n (%) | 888 (67.9) | 111 (60.3) | <0.05* | 367 (61.6) | <0.01* |
| Age (years), median [IQR] | 65.0 [57.0-71.0] | 67.0 [60.0-73.8] | 0.16 | 63.0 [51.0-72.0] | 0.02* |
| Frailty (CFS), median [IQR] | 3 [2.0-3.0] | 3 [2.0-4.0] | <0.001* | 3.0 [2.0-4.0] | <0.001* |
| EQ-5D-5L score, median [IQR] | 0.8 [0.7-0.9] | 0.7 [0.5-0.8] | <0.001* | 0.8 [0.5-0.9] | <0.01* |
| Education level, n (%):  High  Medium  Low | 376 (28.7)  574 (43.9)  358 (27.4) | 25 (13.6)  73 (39.7)  78 (42.4) | <0.001* | 103 (17.3)  258 (43.4)  224 (37.6) | <0.001* |
| Comorbidity (chronic conditions), n (%):  Immunological insufficiency  Malignant hematological disease  Metastasized neoplasm  Cirrhosis  Chronic cardiovascular insufficiency  Chronic respiratory insufficiency  Chronic renal insufficiency | 66 (5.0)  18 (1.4)  58 (4.4)  0 (0)  37 (2.8)  16 (1.2)  21 (1.6) | 28 (15.2)  4 (2.2)  24 (13.0)  2 (1.1)  13 (7.1)  6 (3.3)  13 (7.1) | <0.001*  0.34  <0.001*  0.02*  <0.01*  <0.04*  <0.001* | 44 (7.4)  6 (1.0)  32 (5.4)  3 (0.5)  23 (3.9)  16 (2.7)  15 (2.5) | 0.03* 0.66 0.35 0.03* 0.20  0.03*  0.20 |
| Admission type, n (%):  Planned Surgery  Emergency surgery  Medical | 951 (72.7)  140 (10.7)  217 (16.6) | 104 (56.5)  14 (7.6)  66 (35.9) | <0.001* | 365 (61.2)  136 (22.8)  81 (13.6) | <0.001* |
| APACHE IV score, median [IQR] | 48.0 [38.0-60.0] | 55.0 [42.0-72.8] | <0.001* | 48.0 [37.0-59.3] | 0.67 |
| Mechanically ventilated 24 hours within admission, n (%) | 1020 (78.0) | 120 (65.2) | <0.001* | 400 (67.1) | <0.001* |
| ICU length of stay (days) | 1.0 [1.0-2.0] | 2.0 [1.0-5.0] | <0.001* | 1.0 [1.0-2.0] | 0.26 |

* Statistically significant difference (p<.05) between complete cases and those lost to follow-up due to non-survival and complete cases and those lost to follow-up due to other reasons than non-survival.

*Abbreviations: CFS = Clinical Frailty Scale, EQ-5D-5L = EuroQoL-5D-5L, SF-36 = Short-form 36, APACHE =* Acute physiology and chronic health evaluation
