# Supplementary material for: Optimizing an existing prediction model for quality of life one‐year post‐intensive care unit: An exploratory analysis
Source: Acta Anaesthesiol Scand. 2022 Aug 31;66(10):1228–36. doi: 10.1111/aas.14138 (PMC9804831; doi:10.1111/aas.14138)
Supplement: Supplementary file 5 — Table S5 An overview of the expert‐selected variables for QoL prediction [file AAS-66-1228-s002.docx]

*Additional File 5:* overview of the expert-selected variables for QoL prediction.

**Table S5.** An overview of the expert-selected variables for QoL prediction

| Variable category | Variables | EHR/NICE representation | Model representation |
| --- | --- | --- | --- |
| Demographics | Age, sex*, BMI | Date of birth,  male/female, height in  cm, weight in kg | Age in years at the time of  admission, sex, BMI = weight /  (height)^2^ |
| Clinical measurements | Body temperature, PEEP, FiO_2_, ICP | Order details (e.g. date)  and measured values | Minimum and maximum  during stay, time above or  below threshold value,  standard deviation |
| Laboratory | Hemoglobine, natrium, lactate, glucose | Order details (e.g. date)  and measured values | Minimum and maximum  during stay, time above or  below threshold value,  standard deviation |
| Medication | Noradrenaline, propofol,  midazolam | Order details (e.g. date)  and measured values | Cumulative doses  compensated for LOS and  number of days on medication |
| Stay | LOS, admission timing, admission source* | Admission date,  discharge date,  admission source | LOS in hours, admission within  or outside of office hours,  admission source |
| Monitor | Blood pressure, heart rate | Monitor measurement  details per patient per  variable | Minimum and maximum  during stay, standard  deviation, largest change |
| Other | Tracheostoma, RRT, CVA*, vulnerability*,  comorbidity* | True/false variables,  quantity | Boolean present/absent,  number of times present |

* Also part of the five-feature statistical model.
Abbreviations: EHR = Electronic Health Record; NICE = Dutch Intensive Care Registry; BMI = Body Mass Index; PEEP = Positive End-Expiratory Pressure; FiO_2_ = Fraction of inspired oxygen; ICP = Intracranial Pressure; LOS = Length of Stay; RRT = Renal Replacement Therapy; CVA = Cardiovascular Accident
